# The risk of neurotechnology as an instrument of colonialism

**DOI:** 10.1093/braincomms/fcaf139

**Published:** 2025-04-30

**Authors:** Judy Illes, Makarena Dudley, Lucia Machova Urdzikova, Ioana Podina, Monique Pyrrho

**Affiliations:** Neuroethics Canada, Division of Neurology, Department of Medicine, University of British Columbia, Vancouver, British Columbia, Canada V6T 2B5; Centre for Brain Research, The University of Auckland, Auckland 1023, New Zealand; Department of Neuroregeneration, Institute of Experimental Medicine, Prague 142 20, Czech Republic; Laboratory of Cognitive Clinical Sciences, University of Bucharest, Bucharest 050657, Romania; MINDCARE FOR ALL Association, Bucharest 011761, Romania; International Center for Bioethics and Humanities, University of Brasilia, Brasilia 70910-900, Brazil

## Abstract

Illes et al. focus on strategies and solutions to prevent neurotechnologies from being used to perpetuate colonial power dynamics.

A new path to address neurologic and mental health disorders beyond pharmacological and behavioural interventions has been carved by neurotechnology. The path includes both invasive, intracranial interventions and non-invasive wearable ones. These are systems that record brain signals, stimulate areas of the brain and spinal cord, or both, through open loop systems that feed information to external sources, or closed-loop internal feedback mechanisms. The majority involve various forms of artificial intelligence (AI), and to date, questions about the responsibilities that developers shoulder towards users of these technologies for either medical or non-medical purposes have primarily focused on self-determination, access and human rights. Although the engagement of and with Indigenous Peoples in neuroscience research and innovation has been accelerating over time, little attention has been paid to how advances with neurotechnology may beneficially or dangerously reshape communities whose identities are deeply rooted in traditional beliefs about brain and mind, whose views on neurology and mental health are more about wellness than disease, and who have significant reasons to mistrust Western science and medicine given neglect and abuses of the past.

Positive movement notwithstanding, the slow erosion and invisibilizing of traditional practices is a threat that continues to face Indigenous societies as health technology becomes more advanced and complex. We argue here for the importance of moving from a largely Western lens on living with neurotechnology to an anticipatory and proactive commitment to the future that prevents neurotechnology, where capacity, consent, decision-making, being and believing are core concerns, from becoming an instrument of colonialism.

## Technological colonialism

Technological colonialism describes the use of technoscience to reinforce colonial power dynamics based on strategies of classification, hierarchization, discipline and exploitation that are often justified by ideals of progress or scientific objectivity. Big technoscientific projects, such as neurotechnology, genetics and AI, are presented as Universalist paths to realize the perceived desires about the future of technology and humanity regardless of the diverse cultural contexts they impact. Historically, Universalist ideas of rationality and mental states were tools to delegitimize alternative ways of living, knowing, and thinking, thus reinforcing colonial heriarchies.^1^ Such processes marginalize Indigenous epistemologies and overlook their sustainable approaches to health, well-being and the environment. In doing so, technological colonialism not only marginalizes Indigenous knowledge systems but also threatens autonomy in shaping the future.

## Continua

Since the earliest article in 1963 in which brain, mind and Indigenous populations triangulate, 65 more papers were published through 2020 involving 60 Indigenous communities and authors across 21 countries and 5 continents.^2^ The concepts of wellness, well-being and relationality between individuals and community were prominent reference features in this diverse body of research, consistent with the holistic contextual approach to health where the mind and brain have a harmonious interdependent relationship with other dimensions such as spirituality, family, whole-body and land-based wellness. Many studies focused on mental health and illness, and aging, including the ways that traditional knowledge and biomedical explanations of conditions of the nervous system can co-exist for memory loss in Alzheimer’s disease. Most recently, such co-existence of tradition and biomedicine has been explored in the context of epilepsy, disability, suicide, migration and environmental health.^3^

There have also been positive steps forward in the development of culturally appropriate assessment tools—a form of soft technology—by Indigenous communities for conditions involving cognition and memory, including various ones originating from Australia, Canada, Brazil and New Zealand. These are instruments for detecting dementia in Indigenous populations that have drawn heavily on the knowledge base relative to each Indigenous population. A ‘mate wareware’ app from New Zealand innovators, for example, was designed and released in 2023 to provide information about dementia to Māori families, delivered in a culturally appropriate manner.^4^

By contrast, the field of neurotechnology has largely evolved in the predominant Western paradigm of biological reductionism that focuses on the brain alone with disregard of other systems, human behaviour or custom. By way of example, at the interface of neurotechnology and Indigeneity lies the traditional belief about the special status of the head held by the Māori, the Indigenous People of Aotearoa, New Zealand. The phrase ‘Tuku iho he tapu te upoko’—‘from our ancestors, the head is sacred’—reverberates widely in contemporary Māori society. It is considered inappropriate to touch another person’s head unless invited, and items such as hats or pillows, due to their link with the sacred head, must be treated carefully. Māori customs are practiced at times at the intersection of Western science such as when conducting research on brain diseases or assessing brain functioning, but these traditional practices are the exception and far from routine practice in the field of biological science.

The infusion of technology in health services has gained rapid momentum in the last decade with ubiquitous devices now available for a range of conditions. New technology promises the potential to reduce existing inequities by providing better access to professionals and health information.^5^ For example, wearable EEG-based neurotechnologies and mobile neurofeedback apps were tested to help children with neurodevelopmental disorders to reduce anxiety in school in a low- and middle-income setting. Later studies involved related tests of executive functioning alongside measures of cortisol levels. Access to deep brain stimulation has been studied as an example of the marginalization of remote communities to advanced healthcare that involves neurotechnology, with ensuing recommendations for opportunities possible through telehealth-supported care. Most recently, the ethical, legal and social benefits and risks of portable MRI, which offers new possibilities for neuroimaging in Indigenous and remote settings, including for neurodegenerative, brain injury and neurodevelopmental populations, have come into focus, again with ensuing recommendations for community engagement at the earliest community touchpoints and every stage of research.^6^

Although some Indigenous People have embraced new health and wellness technologies and the benefits they deliver, widespread acceptance is still a long way off as a result of mistrust, a lack of appropriate knowledge translation that limits understanding, and fear of exploitation through loss and exogenous uses of personal data. In parallel, researchers are becoming increasingly mindful of historic patterns of Western scientific enquiry that often involved extractive practices, such as collecting data without consent, appropriating traditional knowledge and disregarding Indigenous perspectives. For both Indigenous communities and researchers, the risk of further marginalizing traditional knowledge by isolating it from, and subjugating it to technology is immense.

## Dimensional protections

Issues of justice related to Indigenous well-being and brain health have been increasingly in focus since the 2021 United Nations Declaration on the Rights of Indigenous Peoples (UNDRIP), and other more recent initiatives focused specifically on neurotechnology in 2024, such as those led by UNESCO (https://unesdoc.unesco.org/ark:/48223/pf0000391444) and the World Health Organization. Despite these efforts, insufficient protections against the risk of neurotechnological colonialism remain. Adherence to the principles of self-determination and free prior and informed consent, as outlined in the UNDRIP, can reduce violence and promote a sustainable society. Eurocentric perspectives, driven by monopolizing colonial power structures, have in the past failed to ensure universal well-being and instead exacerbated inequality and environmental degradation. Indigenous and traditional communities in particular bear the brunt of these failures. Contemporary discourse around neural privacy, Western approaches to consent and exceptional rights for the protection of brain data largely fail to address this point.^7^

There is a need to radically change the way neuroscience engages with Indigenous communities and to focus on decolonization and equity. Neuroscience must value not only biological and cultural diversity but also epistemological diversity. It must leverage Indigenous rights and knowledge and ensure their perspectives are integral to accompanying technological development. Such goals can be achieved through the dimensional protections of access across the clinical and commercial spectrum, attention to issues surrounding the commodification of data, opportunities for digital activism and intellectual property (IP) protections reassessed to be equally meaningful outside Western context (Table 1).

**Table 1:** Dimensional protections against technological colonialism for brain health and wellness

Dimension	Description	Key strategies
**Access**	Indigenous communities face disparities in access to neurotechnologies for brain health and wellness. Misapplication can occur if healthcare providers are not trained to respect Indigenous beliefs. Abuses may occur if information expanding access to products previously unavailable or difficult to procure in the commercial space is misleading.	Dialogue and knowledge exchange about Indigenous practices of brain health and wellness. Equitable and meaningful marketing of brain wellness products. Culturally sensitive interventions for brain health.
**Data commodification and digital farming**	Indigenous communities are often treated as data resources rather than individuals, with their data extracted and commodified without their consent. This is referred to as ‘digital farming.’	Ethical frameworks emphasizing the sovereignty of brain data. Informed consent that provides safeguards against stigmatization based on brain data. Indigenous-controlled brain data repositories to counteract exploitation.
**Scientific and digital activism**	Indigenous communities face economic and technological disparities, limiting their ability to engage in digital activism, particularly within neurotechnological spaces.	Promotion of Indigenous involvement and prominence in all phases of the development of neurotechnologies, including in academic spaces. Investments in digital infrastructure, such as through localized digital learning hubs, so that Indigenous People are engaged from the earliest phases of neurotechnology innovation. AI-assisted platforms that are accessible and tailored to Indigenous needs for effective advocacy for brain health and wellness.
**Intellectual property**	Current models are insufficient to protect Indigenous knowledge, which is collectively owned and holistic in nature.	IP models for brain data that respect Indigenous knowledge and its cultural context. Profit-sharing that is genuinely mutually beneficial. Transparent agreements for the use of Indigenous knowledge about brain health and wellness. Legal protection frameworks that ensure that Indigenous knowledge is safeguarded.

### Access

The co-existence of neurotechnologies and the relief of conditions affecting the CNS must be guided by the rights of Indigenous Peoples to their land, craft and traditional practices. Disparities in access lead to poor outcomes and misapplication of treatments if healthcare providers are not trained to respect Indigenous beliefs. Requirements to leave communities to pursue care further compounds these challenges. These strategies must span the full range of providers in health care. In the commercial space for the promotion of wellness, questions pertain both to material acquisition of Western products in addition to access to information and marketing practices that are non-misleading.

### Data commodification and digital farming

In colonial contexts, data are objects from which Western society extracts knowledge and value. In digital colonialism discussions, the practice of treating populations as resources of data is called digital farming, as people are treated as stock that produce a kind of commodity: data.^8^ To address this, ethical frameworks must emphasize data sovereignty for Indigenous communities that ensure the rights of Indigenous Peoples and tribes to govern the ownership, collection and protection of their own data (https://fnigc.ca/ocap-training/) and that results benefit their communities. Strategies require informed consent processes that align with Indigenous values, and provide transparency about how data will be utilized, stored and shared. As for other forms of data, but more acutely for the brain and mind, Indigenous-controlled repositories will guard against the exploitation of neurological data under the guise of scientific advancement.

### Scientific and digital activism

Indigenous communities face economic and technological disparities limiting their ability to engage in digital activism and related self-governance. Digital activism is seen throughout various movements advocating for Indigenous rights, from land sovereignty to cultural preservation. To address these disparities, localized community digital learning hubs can bridge the gap by providing Indigenous communities with the resources and training necessary to engage with digital tools.^9,10^ AI also holds great potential for Indigenous advocacy if tailored for purposes envisioned by Indigenous Peoples and then trained by datasets that are relevant and sourced ethically. However, like with health care, without equitable access, AI will further deepen digital divides. Investments in digital infrastructure and literacy are essential to empower Indigenous activists, allowing them to use AI and digital tools for language translation and access published scientific literature, and in community exchange, advocacy and policy reforms.

### Intellectual property

Current IP protection models are not suitable to protect Indigenous knowledge, which is holistic and collectively owned. We acknowledge that this is a ubiquitous problem that does not pertain to the neuro space only. Alternative models for all innovation should be developed to maintain the cultural and physical environments that generate knowledge with respect to and outside inventions surrounding brain and mental health. In one partnership between Mt Romance, Aveda and the Kutkabubba community from Western Australia, for example, the use of Indigenous knowledge was reported to provide reciprocal economic benefits and recognition. Some scholars are critical of such a profit-sharing approach, however, as it is viewed to be exploitive rather than empowering of vulnerable people.^8^ Another example is the recent amendment to South African laws, particularly the Intellectual Property Laws Amendment Act 28 of 2013 that recognizes and protects Indigenous knowledge and establishes a National Council and National Databases for ensuring that any use of such knowledge involves community consent and benefit-sharing.

## Summary

Technological colonialism can be mitigated in global brain health and wellness with a reversal of existing historical power imbalances and prevention of future ones. Science must fundamentally change its relationship with Indigenous knowledge if it hopes to move away from colonialism and implement a pluralistic approach to knowledge that respects Indigenous sovereignty, intellectual and cultural beliefs, practices and resources. Knowledge sharing can no longer be contingent upon economic power or evaluated solely based on current scientometric parameters. Dominant conversations about mental privacy are distractions from fundamental injustices that have existed since time immemorial and that must be resolved before such more nuanced topics are relevant and can be addressed fairly. Focused approaches to global standard-setting will prevent the misuse of technology for brain health and wellness in ways that previously perpetuated colonial power dynamics, and ensure that these technologies are developed and deployed in ways that respect the rights and dignity of all people.
